# An EEG-based fatigue detection framework integrating interpretable feature selection and efficient temporal modeling

**DOI:** 10.3389/fnins.2026.1903252

**Published:** 2026-07-31

**Authors:** Xiaohong Jiang, Lili Chen, Xin Zhao, Tao Chen, Mingyang Chu, Hao Wu, Xinbo Gao

**Affiliations:** 1 College of Traffic and Transportation, Chongqing Jiaotong University, Chongqing, China; 2 School of Information Science and Engineering, Chongqing Jiaotong University, Chongqing, China; 3 School of Computer Science and Technology, Chongqing University of Posts and Telecommunications, Chongqing, China; 4 School of Mechatronics and Vehicle Engineering, Chongqing Jiaotong University, Chongqing, China; 5 The First Affiliated Hospital of Chongqing Medical University, Chongqing, China; 6 Xidian University, Xi’an, China

**Keywords:** attention mechanism, channel selection, electroencephalogram (EEG), fatigue detection, interpretable feature selection, lightweight model

## Abstract

**Introduction:**

Fatigue is a critical factor contributing to operational errors, traffic accidents, and psychological disorders. A major challenge in current fatigue detection lies in the difficulty of clearly identifying fatigue-related neural channels in the brain, which hinders the accurate extraction of effective features from high-dimensional electroencephalography (EEG) signals.

**Methods:**

This study proposes the AXELLSTM fatigue detection framework, an EEG-based cross-subject fatigue detection framework that integrates interpretable feature selection with efficient temporal modeling. Specifically, a nested leave-one-subject-out (LOSO) validation strategy was adopted to evaluate cross-subject generalization and reduce potential data leakage during feature and channel selection. Within each LOSO fold, ANOVA and XGBoost were first used to identify discriminative fatigue-related features, followed by an L1-regularized sparse selection model to estimate channel importance. Furthermore, an EfficientLSTM model was designed by integrating a feature compression module, bidirectional long short-term memory network, channel attention, and temporal attention mechanisms to capture sequence-level EEG temporal representations.

**Results:**

The results showed that eight features, including mean, RMS, zero-crossing rate, gamma power, beta power, standard deviation, variance, and peak-to-peak value, were consistently selected across all LOSO folds. In addition, 18 EEG channels, including F8, AF8, TP8, F2, Fp1, F4, FT10, F6, Fz, P7, FT9, O1, C5, FT8, CP4, F3, FC6, and P3, were stably retained, suggesting that fatigue-related EEG information is distributed across frontal, frontopolar, frontotemporal, parietal, and occipital regions. Under the LOSO protocol, EfficientLSTM achieved an accuracy of 0.7454 ± 0.2147, balanced accuracy of 0.7436 ± 0.2139, and Macro-F1 of 0.7143 ± 0.2452, with 0.1959 M parameters, a model size of 0.7474 MB, and 3.7069 M FLOPs. Compared with the baseline models, EfficientLSTM obtained the highest average Macro-F1 and showed a favorable balance between cross-subject recognition performance, model complexity, and temporal modeling capability.

**Discussion:**

These findings indicate that the proposed AXELLSTM framework provides an interpretable and computationally efficient solution for EEG-based fatigue detection and offers potential support for the development of portable fatigue monitoring systems.

## 1 Introduction

Fatigue, as a complex multidimensional psychophysiological state, is increasingly recognized as a significant public health issue, a major contributor to safety incidents and economic burdens, and a critical factor leading to operational errors, traffic accidents, and psychological disorders (Doran et al., 2001). Accurate and objective identification of fatigue is therefore of great significance for ensuring personnel safety in high-risk industries, reducing fatigue-related traffic accidents, and improving the clinical diagnosis and management of fatigue-associated diseases. In recent years, increasing research attention has been directed toward the exploration of diverse physiological and behavioral indicators for fatigue detection. However, conventional fatigue assessment methods predominantly rely on subjective self-report scales, which are inherently influenced by individual variability, recall bias, and participants’ subjective willingness, thereby substantially limiting their reliability and validity (Krupp et al., 1989). In practice, non-physiological signals, such as facial expressions (Ahmed et al., 2021) and speech signals, satisfy several of the aforementioned requirements for fatigue detection. Nevertheless, these signals are highly susceptible to voluntary control and intentional manipulation, rendering them vulnerable to forgery and consequently limiting their reliability and robustness in fatigue monitoring applications. By contrast, physiological signal measurement techniques provide a more objective, stable, and accurate means of capturing alterations in the body’s physiological state, thereby offering greater potential for reliable fatigue assessment (Adão Martins et al., 2021).

In recent years, driven by advancements in the miniaturization and portability of physiological sensing technologies—such as electroencephalography (EEG) (van Weelden et al., 2022), electrocardiography (ECG) (Guo D. et al., 2025), and electromyography (EMG) (Naim et al., 2022)—as well as progress in signal processing and machine learning algorithms, physiological-signal-based fatigue recognition has attracted increasing attention. Among these modalities, ECG and EMG have been widely used because of their relatively simple acquisition procedures and their ability to reflect autonomic nervous regulation and muscular activity, respectively. However, these signals mainly characterize peripheral physiological responses, which can be easily affected by factors such as physical activity, posture, respiration, emotional fluctuations, and muscle tension. Therefore, they may provide indirect or nonspecific indicators when the target is cognitive or mental fatigue. In contrast, EEG directly records the electrical activity of the central nervous system and provides high temporal resolution for capturing dynamic changes in brain function during fatigue development (Yin and Zhang, 2018). Mental fatigue is closely associated with alterations in neural oscillations, especially changes in frequency-specific EEG rhythms and region-dependent brain activity (Tanaka et al., 2014). Therefore, EEG is particularly suitable for detecting fatigue states that originate from cognitive load, sustained attention decline, and reduced neural regulation. These advantages make EEG a more direct and physiologically interpretable modality for fatigue classification compared with peripheral physiological signals such as ECG and EMG.

Although EEG signals exhibit considerable potential for fatigue state recognition, existing studies predominantly rely on high-density EEG acquisition paradigms with fixed channel configurations, while insufficiently accounting for inter-individual variability in neural responses and task-specific contextual differences (Lawhern et al., 2018). Such electrode configurations not only result in the acquisition of large volumes of redundant data, thereby increasing computational burden and hardware complexity, but may also degrade the discriminative performance of fatigue recognition models by introducing noise components unrelated to fatigue (Guo H. et al., 2025). Therefore, it is essential to extract features that are truly and strongly associated with fatigue states from high-dimensional EEG data. On this basis, an effective channel selection strategy should be designed to identify a minimal yet maximally discriminative subset of electrodes. Such an approach can significantly improve computational efficiency and model robustness, while also providing a crucial foundation for the development of lightweight, personalized, and portable fatigue monitoring systems suitable for real-world applications.

To address the aforementioned challenges, this study proposes an EEG-based fatigue detection framework named AXELLSTM, which integrates interpretable feature selection with efficient temporal modeling. The AXELLSTM framework employs rigorous statistical analysis combined with machine learning-based feature selection methods to identify a minimized channel-feature subset with optimal discriminative capacity. Concurrently, an EfficientLSTM model is designed to accurately detect fatigue states, substantially enhancing the computational efficiency and interpretability of EEG-based fatigue detection models. These contributions collectively establish a novel methodological foundation for precise and efficient fatigue state recognition.

The core contributions of this work lie in:

(1) Systematically identify the EEG channels and key features most strongly associated with fatigue states, thereby precisely localizing the brain regions that exhibit high specificity and high sensitivity for fatigue recognition.

(2) By integrating a feature compression module, bidirectional long short-term memory networks (BiLSTM), and a dual-attention mechanism that jointly models channel-wise and temporal information, an efficient LSTM architecture (EfficientLSTM) is constructed, enabling precise identification and robust representation of fatigue states.

(3) Extensive experimental results systematically demonstrate that optimized channel selection can substantially reduce the complexity of EEG acquisition systems while maintaining high detection accuracy, thereby providing a crucial prerequisite for the development of practical and wearable fatigue monitoring technologies.

## 2 Related work

This section provides an overview of recent research on EEG-based fatigue detection methods, including channel selection techniques for EEG signals and approaches for fatigue detection using EEG signals.

### 2.1 Channel selection methods based on EEG signals

Tenev et al. (2014) employed a forward feature selection strategy to identify significant features for attention-deficit/hyperactivity disorder (ADHD) classification. Khoshnoud et al. (2018) quantified the chaotic nonlinear dynamics of EEG signals using the multifractal singularity spectrum, the largest Lyapunov exponent, and approximate entropy, subsequently applying principal component analysis (PCA) for dimensionality reduction to extract strongly correlated features. Wang et al. (2023) processed EEG signals acquired from the frontal FP1 and FP2 electrodes by extracting multiscale entropy and autocorrelation coefficient features, while employing common spatial patterns (CSP) to derive variance-based EEG features, resulting in a classification accuracy of 95.94%. Liu X. et al. (2021) introduced a fatigue detection framework leveraging the non-hairy region (NHB) approach, aiming to facilitate practical driving fatigue monitoring by reducing the number of EEG channels within the NHB region and thereby achieving more efficient EEG acquisition and utilization. Zhou et al. (2023) systematically evaluated 26 feature sets using eight machine learning algorithms to identify the optimal feature–algorithm combinations for fatigue detection. However, this exhaustive search strategy proved computationally intensive and failed to achieve effective data dimensionality reduction. Liu Q. et al. (2021) selected eight optimal EEG electrode channels and extracted fused features for fatigue detection; however, the resulting classification accuracy for test subjects did not exhibit a substantial improvement. Similarly, Zhang et al. (2023) proposed a method for identifying an optimal feature subset, yet the achieved accuracies across eight classification models remained below 80%.

### 2.2 Fatigue detection methods based on EEG signals

Deng et al. (2020) proposed a deep sparse shrinking autoencoder network to learn localized fatigue-related features, which demonstrated superior performance in identifying pilots’ mental fatigue states on the evaluated dataset. Li et al. (2024) developed a CWSTR-Net for fatigue detection, which surpassed existing EEG-based models by achieving a remarkable 97.23% accuracy on the SEED_VIG dataset. Liu et al. (2024) engineered a shallow hybrid network, 1DCNN-BiLSTM, to extract spatial features across different channels and forward-backward temporal features from EEG signals. Experiments conducted on the DEAP and DREAMER datasets achieved a recognition accuracy of over 90%. Geng et al. (2025) introduced a novel preprocessing and feature extraction paradigm combining EEMD and FastICA, which effectively mitigates physiological artifacts to ensure signal integrity. Empirical evaluations confirm that, unlike limited single-feature methods, their comprehensive multi-feature fusion approach excels at extracting latent fatigue-related information, resulting in a marked improvement in the classification performance of driver fatigue detection systems.

In recent years, EEG-based cognitive state estimation has increasingly emphasized joint spatial–temporal representation learning and model optimization. Early studies have begun to focus on the spatial distribution patterns and temporal dynamic characteristics of multi-channel EEG signals. For example, Gao et al. (2019) proposed ESTCNN, which automatically learns spatial correlations and temporal dependencies from EEG signals through a spatial–temporal convolutional structure for driver fatigue evaluation. Subsequently, Peng et al. (2024) proposed 3D-STCNN, which integrates three-dimensional spatial EEG features with a spatial–temporal convolutional network to enhance the representation of spatial structures in driving fatigue detection Sun et al. (2025) further proposed SGL-Net, which combines a spectral group-guided mechanism with a lightweight CNN and designs a hardware system tailored for frontal EEG acquisition, reflecting the trend of EEG fatigue detection toward both high-accuracy modeling and lightweight rapid deployment. Building on these advances Chen et al. (2026) adopted a hybrid CNN-BiMGRU-MHAM architecture to jointly extract spatial features, temporal dependencies, and attention-enhanced discriminative representations, and proposed an EEG-based pilot fatigue detection framework that integrates brain power mapping with optimized deep learning. Although EmoDLNet was originally developed for EEG-based emotion recognition rather than fatigue detection (Geda et al., 2026), it provides a representative example of end-to-end multi-scale spatio-temporal deep learning for EEG-based cognitive state estimation, further demonstrating the importance of joint spatial–temporal representation learning in EEG signal analysis.

In recent years, a variety of EEG channel selection strategies and fatigue recognition models have been proposed, leading to notable advances in channel reduction and improvements in computational efficiency. However, existing approaches often suffer from inherent trade-offs: methods that emphasize channel selection can effectively reduce system complexity but frequently do so at the expense of recognition accuracy, whereas models that pursue high detection precision typically depend on a large number of EEG channels and thus fall short of achieving truly lightweight designs. To surmount these hurdles, we introduce a synergistic framework that jointly optimizes feature and channel selection with efficient temporal modeling. By designing a novel fatigue recognition model, the proposed approach substantially reduces the number of EEG channels while maintaining, and in some cases improving, fatigue detection accuracy. Consequently, this framework provides a promising direction for the development of fatigue monitoring systems that simultaneously achieve lightweight implementation and high precision.

## 3 Materials and methods

### 3.1 Data description

We recruited 16 participants from Chongqing Jiaotong University and Chongqing Medical University to take part in the experiment. This study was conducted in accordance with the Declaration of Helsinki and approved by the Institutional Review Board (IRB) of the First Affiliated Hospital of Chongqing Medical University (protocol code: (2024) Scientific Ethics Review (CY2024-027-01), approved on 20 May 2024). Informed consent was obtained from all participants involved in the study (Version V1.2/2024.05.01). The average age of all participants was 23 ± 1.2 years. A 64-channel actiCHamp EEG acquisition system manufactured by BP Germany (Beijing Huaruibao Technology Co., Ltd., 2023) was used, and data were recorded with a 64-channel EEG cap at a sampling rate of 500 Hz. The experimental paradigm employed the classic Psychomotor Vigilance Test (PVT) and the 2-back task. For the PVT, participants were instructed to fixate on a white “+” sign displayed on the screen. They were required to press a button as quickly as possible whenever the “+” sign turned red. In the 2-back task, participants needed to continuously maintain and update information in working memory throughout a sequence of serially presented letters. Upon the appearance of each new letter, they had to judge whether it matched the letter presented two positions earlier; if a match was identified, they were required to press a response button.

Initial processing of the EEG recordings was conducted using the EEGLAB toolbox in MATLAB. The specific steps included: (1) Initially using FCz as the reference electrode, serving as the baseline for all signal channels; (2) Applying a re-referencing technique using the bilateral mastoids (TP9 and TP10) as the new reference electrodes; (3) Applying a 0.1–100 Hz bandpass filter; (4) Processing the EEG signals using Independent Component Analysis (ICA); (5) Performing automatic artifact removal; (6) Segmenting the EEG data from the 3-min PVT, REST, and 2-back task stages into 2-s epochs for analysis.

A total of 16 participants were included in this experiment. To more rigorously evaluate the cross-subject generalization ability of the proposed model on unseen participants, a leave-one-subject-out cross-validation (LOSO-CV) strategy was adopted for model training and testing. Specifically, in each fold, all samples from one participant were used as the independent test set, while all samples from the remaining 15 participants were used as the training set. This procedure was repeated 16 times so that each participant was used once as the held-out test subject. The final model performance was reported as the mean and standard deviation across the 16 LOSO folds, thereby providing a more comprehensive evaluation of inter-subject stability and generalization ability.

To avoid potential data leakage during feature selection, ANOVA, XGBoost, and L1-regularized logistic-regression-based sparse feature-channel selection were performed only on the training participants within each LOSO fold. The L1-regularized model follows the sparsity-inducing principle of LASSO and was used to estimate the contribution of each feature-channel variable within the current training fold. The held-out test participant was not involved in feature selection, channel selection, preprocessing parameter estimation, model training, or hyperparameter tuning. The EfficientLSTM model was then trained using the selected features and channels from the current training fold and evaluated on the corresponding held-out participant. Based on the fatigue induction paradigm and the behavioral validation results reported in the Results section, PVT1, MID, and PVT2 were operationally defined as the alert, intermediate, and fatigue states, respectively.

#### 3.1.1 Subjective fatigue assessment

Subjective fatigue was assessed before and after the fatigue induction task using an eight-level self-rating scale designed with reference to commonly used subjective sleepiness and fatigue scales, such as the Karolinska Sleepiness Scale (KSS) (Åkerstedt and Gillberg, 1990), as shown in Table 1. The scale ranged from “extremely relaxed” to “very fatigued and difficult to stay awake,” with higher scores indicating a higher subjective fatigue level. Participants were instructed to select the option that best matched their current state before and after the experiment. For statistical analysis, the eight response options were converted into numerical scores ranging from 1 to 8. The pre- and post-induction subjective fatigue scores were then compared to evaluate whether the continuous 2-back task increased perceived fatigue. This subjective assessment, together with PVT reaction-time performance, was used to support the operational definition of fatigue states.

**TABLE 1:** Subjective fatigue self-rating scale and quantified scores.

Score	Self-rating option	Reference state
1	Extremely relaxed	Alert (PVT1)
2	Relatively relaxed	
3	Slightly relaxed	
4	Neither relaxed nor fatigued	Intermediate (MID)
5	Slightly fatigued	
6	Fatigued but able to stay awake	Fatigue (PVT2)
7	Fatigued and making effort to stay awake	
8	Very fatigued and difficult to stay awake	

#### 3.1.2 PVT test

After completing the subjective fatigue assessment, participants performed the Psychomotor Vigilance Task (PVT). The procedure of the PVT was as follows. Participants were instructed to continuously fixate on a white “+” symbol presented at the center of the screen. When the symbol changed from white to red, they were required to press the response key as quickly as possible. Reaction time was defined as the time interval between the color change of the stimulus and the participant’s key press. In general, a shorter reaction time indicates better vigilance and sustained attention, whereas prolonged reaction time or missed responses may suggest reduced vigilance, impaired response ability, and increased fatigue. In this study, two PVT tests were conducted before and after the fatigue induction task, namely PVT1 and PVT2. PVT1 was performed before the continuous 2-back fatigue induction task and was used to represent the baseline alert state, whereas PVT2 was performed after the continuous 2-back fatigue induction task and was used to evaluate fatigue-induced behavioral changes. For each PVT test, the total reaction time was extracted as a behavioral indicator and calculated using Equations 1, 2, as follows:


$$T\,R\,T=\overset{n}{\underset{i=1}{\sum }}R\,T_{i}$$ (1)

where *RT*_*i*_ denotes the reaction time of the *i*-th valid response, and *n* represents the total number of valid responses. Furthermore, the change in total reaction time before and after fatigue induction, denoted as △*TRT*, was defined as:


$$△\,T\,R\,T=T\,R\,T_{P\,V\,T_{2}}-T\,R\,T_{P\,V\,T_{1}}$$ (2)

When △*TRT*, it indicates that participants exhibited slower response speed and reduced vigilance after fatigue induction. In the Results section, a paired statistical test was further performed to compare the total reaction times between PVT1 and PVT2. Together with changes in subjective fatigue ratings, this analysis was used to validate the effectiveness of the fatigue induction paradigm and to support the rationale for defining PVT1, MID, and PVT2 as the alert, intermediate, and fatigue states, respectively.

### 3.2 Fatigue detection framework

**FIGURE 1:**
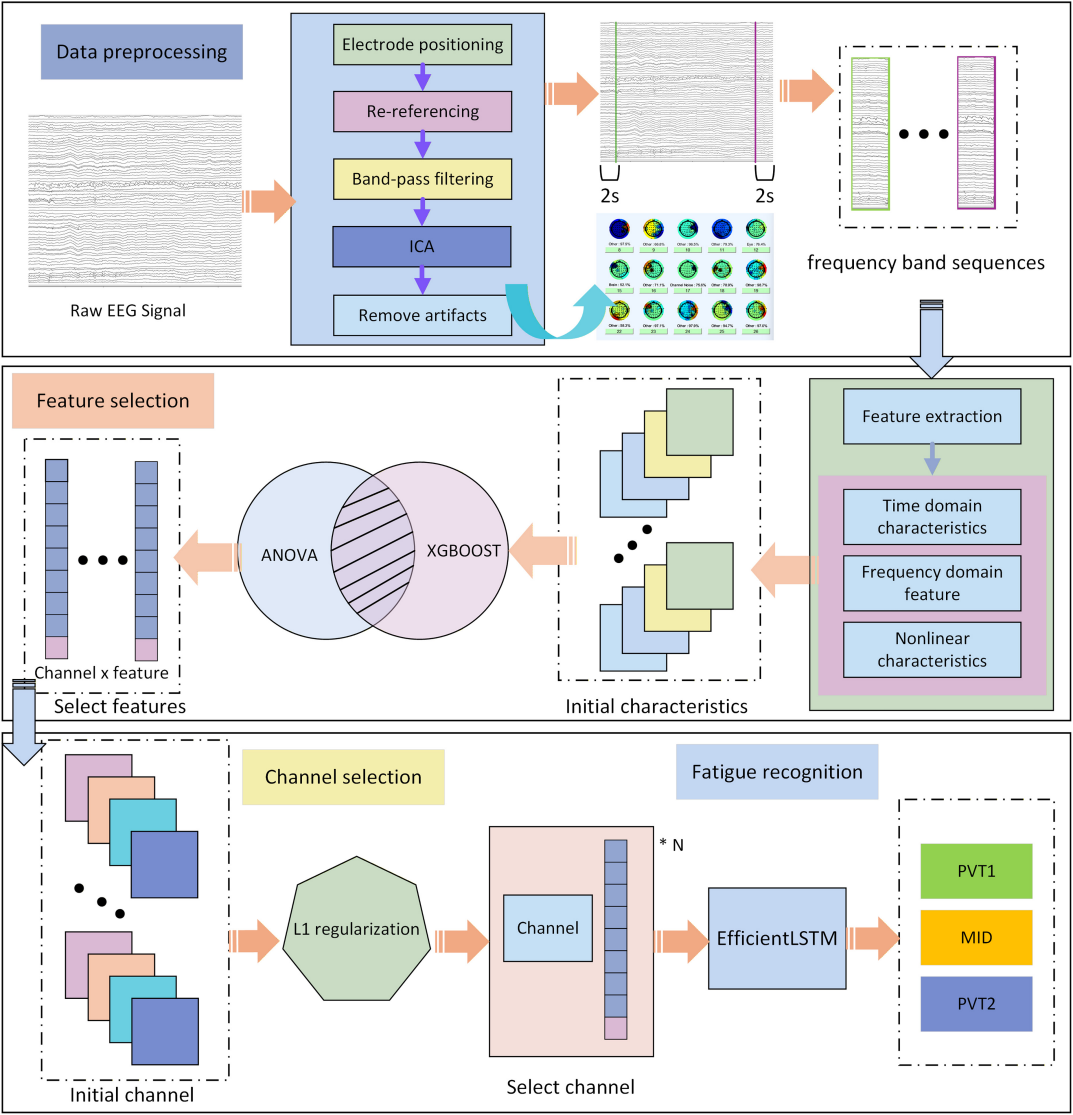
Flowchart of the three-step progressive feature selection strategy.

To simultaneously boost model efficiency and interpretability, we propose AXELLSTM, a fatigue detection framework for broad application scenarios that integrates interpretable feature selection with efficient temporal modeling. We executed a progressive three-step feature selection strategy that systematically distills the optimal feature subset. Subsequently, based on the selected channels, we utilized our proposed EfficientLSTM model for fatigue detection. Figure 1 illustrates the detailed workflow of this strategy. To avoid potential data leakage, all feature selection, channel selection, missing-value imputation, normalization, and model training procedures were embedded within a nested leave-one-subject-out (LOSO) validation framework. In each LOSO fold, one participant was held out as the independent test subject, while the remaining participants were used for feature screening, channel selection, and model training. First, we employed ANOVA to determine which candidate features exhibited statistically significant differences. Features exhibiting a significance level of *p* < 0.05 were retained, thereby filtering out variables with insufficient discriminatory power—a methodological approach validated in prior studies (Zhang et al., 2023). Second, an XGBoost-based feature evaluation procedure was conducted within the training fold to further assess the discriminative capability of the features retained after ANOVA screening. Specifically, each retained feature type was evaluated using an XGBoost classifier with inner cross-validation, and features showing sufficient classification ability were preserved for subsequent channel-level selection. In the final stage, an L1-regularized sparse selection model was applied to estimate the contribution of each EEG channel. By leveraging its L1 regularization property, the sparse selection model induces sparsity in the feature space by suppressing less informative variables while retaining discriminative fatigue-related information, thereby yielding a refined feature set characterized by high discriminative capacity and enhanced interpretability. This approach effectively integrates the complementary advantages of statistical hypothesis testing, machine learning, and regularization methodologies. It not only maintains robust classification performance but also mitigates overfitting risks, thereby establishing a transparent feature basis for subsequent interpretation of underlying physiological or cognitive mechanisms.

#### 3.2.1 Multi-stage interpretable channel selection

As shown in Table 2, a total of 19 EEG features were extracted from the preprocessed EEG signals of the 16 participants, including eight time-domain features, ten frequency-domain features, and one nonlinear feature. To avoid potential data leakage caused by performing feature selection before cross-validation, the feature and channel selection procedures were embedded into a nested leave-one-subject-out (nested LOSO) cross-validation framework. In each LOSO fold, one participant was completely held out as the independent test subject, while the remaining 15 participants were used as the training set. All feature screening, channel selection, missing-value imputation, and normalization procedures were performed only on the training subjects of the current fold. The held-out test subject was not involved in any step of feature selection, channel selection, preprocessing parameter estimation, or model training. In the first stage, one-way analysis of variance (ANOVA) was independently conducted for each original feature type within the current training fold to examine whether significant differences existed among the three fatigue states. The significance threshold was set to (*p* < 0.05). This step was designed to remove redundant variables with limited inter-state discriminative ability while retaining candidate features with potential fatigue-related statistical relevance. In the second stage, to further quantify the classification capability of each statistically significant feature, an XGBoost classifier was employed as the feature evaluation model. A stratified five-fold cross-validation strategy was performed within the current training fold. For each candidate feature type, all channel-wise feature columns corresponding to that feature were first standardized using parameters estimated only from the training data and then individually fed into a lightweight XGBoost model for evaluation. The mean classification accuracy of each single-feature model across the five inner cross-validation folds was subsequently calculated. If the average single-feature classification accuracy of a given feature exceeded the preset threshold of 0.60, the feature was considered to have sufficient independent discriminative ability and was retained for subsequent channel selection.

**TABLE 2:** Full list of extracted EEG features before feature selection.

Feature category	Extracted features
Time-domain features	mean, std, var, rms, skewness, kurtosis, peak to peak, zero crossing rate
Frequency-domain features	delta power, delta relative power, theta power, theta relative power, alpha power, alpha relative power, beta power, beta relative power, gamma power, gamma_relative_power
Nonlinear feature	Sample entropy

To comprehensively evaluate the contribution of each EEG channel to fatigue recognition, this study designed a composite channel importance score based on the sparse coefficients obtained from L1-regularized logistic regression. Although the model used here is a classification model rather than ordinary least-squares LASSO regression, it follows the same L1-induced sparsity principle as LASSO, where less informative variables are shrunk toward zero. Therefore, it can be regarded as a LASSO-type sparse selection model. Following the L1-based feature selection principle introduced by Tibshirani (2011), and inspired by feature selection studies emphasizing both feature relevance and selection stability (Bolón-Canedo et al., 2013), the proposed channel importance score integrates two complementary indicators: the average absolute coefficient magnitude of features within each channel and the support ratio of non-zero coefficients within that channel.

For a given EEG channel *c*, let *I_c_* denote the set of feature indices belonging to this channel, and *n*_*c*_ = |*I*_*c*_| denote the number of features in *I_c_*. The average absolute coefficient magnitude of channel *c* is calculated according to Equation 3, as follows:


$$A_{c}=\frac{1}{n_{c}}\,\underset{i\in I_{c}}{\sum }\left|\beta_{i}\right|$$ (3)

Where β_*i*_ denotes the coefficient of the *i*-th feature obtained from the L1-regularized model.

The maximum average coefficient magnitude among all channels is calculated according to Equation 4, as follows:


$$A_{\max}=\max_{c}A_{c}$$ (4)

Where *c* = 1,2,…, *C* and *C* is the total number of EEG channels.

The support ratio of channel c is calculated according to Equation 5, as follows:


$$R_{c}=\frac{1}{n_{c}}\underset{i\in I_{c}}{\sum }1\left(|\beta_{i}|>\varepsilon \right)$$ (5)

where 1(⋅) is the indicator function (equals 1 if the condition holds, 0 otherwise), and ε is a small positive threshold used to determine whether a coefficient is non-zero (set to ε = 10^−10^ in this study).

The final composite importance score of channel *c* is calculated according to Equation 6, as follows:


$${\mathrm{importance}}_{\_}\,s\,{\mathrm{core}}_{c}=\alpha \times \left(\frac{A_{c}}{A_{\max}+\delta }\right)+\left(1-\alpha \right)\times R_{c}$$ (6)

Where α ∈ [0,1] is a weighting parameter. In this study, α was set to 0.7 to emphasize the contribution strength reflected by coefficient magnitude, and δ = 10^−10^ was used to avoid division by zero.

#### 3.2.2 EfficientLSTM

**FIGURE 2:**
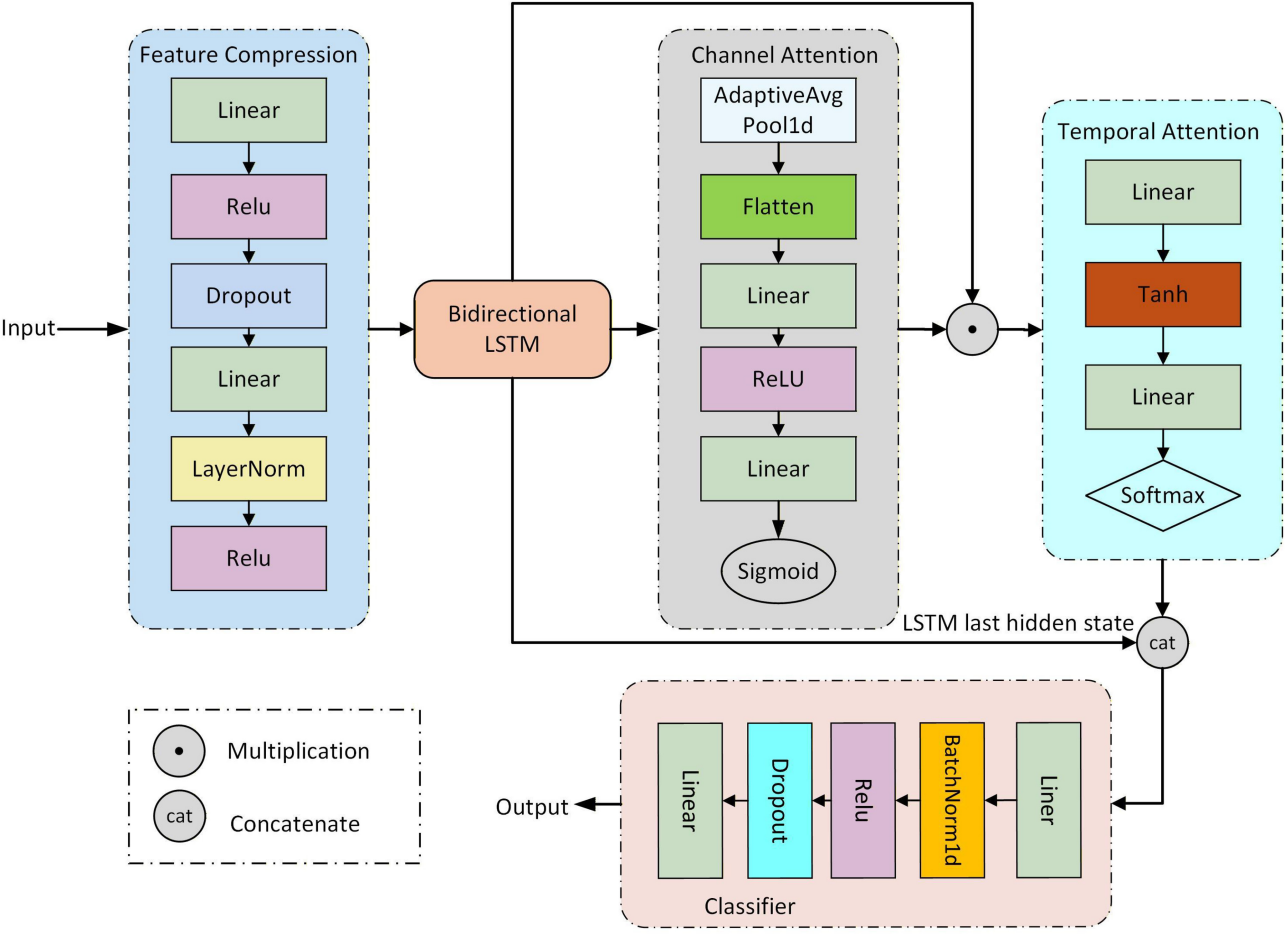
EfficientLSTM model framework.

To effectively capture the temporal dynamics of EEG signals during fatigue progression and improve classification performance, we propose an efficient fatigue recognition model, termed EfficientLSTM, which integrates feature compression, BiLSTM, and a dual-attention mechanism, as schematically depicted in Figure 2, its parameter composition is summarized in Table 3. The model initially compresses the original high-dimensional input features and applies nonlinear transformations via a feature compression module, thereby reducing redundant information and aligning the dimensionality with the requirements of subsequent sequential modeling. The compressed feature sequence is then fed into a BiLSTM layer. This architecture captures both forward and backward contextual dependencies within the time series, yielding more comprehensive temporal representations. The output dimension of this layer is twice the hidden state size, i.e., 128. To further augment the model’s capacity to emphasize informative feature channels and salient temporal segments, a dual-attention mechanism—comprising Channel Attention and Temporal Attention modules—is incorporated following the BiLSTM layer (Duyar et al., 2024). The Channel Attention module is inspired by the Squeeze-and-Excitation (SE) architecture (Hu et al., 2018), which learns importance weights for each feature channel by applying global average pooling along the temporal dimension. Following normalization via a sigmoid activation function, these weights are element-wise multiplied with the original BiLSTM output, thereby achieving adaptive channel-wise feature recalibration. The recalibrated features are subsequently processed through the Temporal Attention module to derive a globally contextualized temporal aggregation feature vector *f*_*temp*_ ∈ *R*^128^. This weighted representation is then concatenated with the final hidden state of the LSTM, yielding a joint embedding with a dimensionality of 256. The combined vector is fed into a fully connected classification head, which incorporates batch normalization, ReLU activation, and dropout regularization, to enable efficient and robust fatigue recognition.

**TABLE 3:** Parameter composition of the proposed EfficientLSTM model.

Module	Main operation	Input → output	Key parameters
FCM	Feature compression	D → D/2→64	Linear-ReLU-Dropout-Linear-LayerNorm-ReLU
BiLSTM	Bidirectional temporal feature extraction	64 → 128	2-layer BiLSTM, hidden size = 64
CA	Channel-wise feature recalibration	128 → 128	SE-style attention, reduction ratio = 16
TA	Temporal importance weighting	128 → 128	MLP-based temporal attention
Classifier	Fatigue state classification	256 → 3	Linear-BN-ReLU-Dropout-Linear

D denotes the input feature dimension of each feature matrix. Specifically, D is 19 × 62 for matrix A, 8 × 62 for matrix B, and 8 × 18 for matrix C.

In the Feature Compression module (FCM), the first linear layer reduces the input dimension from din to din/2, and the second linear layer further projects it to the LSTM hidden unit dimension of 64. ReLU activation, moderate Dropout regularization, and Layer Normalization are applied between these layers to mitigate overfitting and facilitate convergence. The detailed computational procedures are presented in Equations 7–9.


Z1=Re⁢L⁢U⁢(W1⁢X+b1) (7)

$$Z_{2}=\mathrm{Dropout}\,\left(Z_{1}\right)$$ (8)

Xred=L⁢a⁢y⁢e⁢r⁢N⁢o⁢r⁢m⁢(Re⁢L⁢U⁢(W2⁢Z2+b2)) (9)

Where: the input sequence is *X* ∈ *R*^*B* × *T* × *d*_*in*_^, *d*_*in*_ is the original feature dimension, where $W_{1}\in R\,\frac{d_{\mathrm{in}}}{2}\times d_{\mathrm{in}}$notes the weight matrix of the first layer, *b*_1_ represents the corresponding bias vector, $W_{2}\in R^{h\times \frac{d_{\mathrm{in}}}{2}}$ is the weight matrix of the second layer, *b*_2_ represents the corresponding bias vector, and *h* is the number of LSTM hidden units.

BiLSTM extends the conventional LSTM architecture by incorporating two independent hidden layers: One captures temporal dependencies in the forward direction, while the other models them in the reverse direction. The output at each time step is synthesized from the hidden states of both directions, typically through concatenation or a learned weighted combination. The detailed computational procedures are presented in Equations 10–13.


$$\vec{h_{t}}=L\,S\,T\,M\,f\,w\,d\,\left(x_{t},\vec{h_{t-1}}\right)$$ (10)

$$\overset{\leftarrow }{h_{t}}=L\,S\,T\,M_{b\,w\,d}\,\left(x_{t},\overset{\leftarrow }{h_{t+1}}\right)$$ (11)

$$h_{t}=\left[\vec{h_{t}},\overset{\leftarrow }{h_{t}}\right]\in R^{2\,h}$$ (12)

$$H=\left[h_{1},h_{2},\ldots ,h_{T}\right]\in R^{B\times T\times 2\,h}$$ (13)

Channel attention (CA) learns importance weights for each feature channel by performing global average pooling along the temporal dimension and recalibrates the LSTM output via channel-wise scaling. Initially, global average pooling is applied over the entire temporal sequence to generate a compressed channel-wise statistic vector z, which encapsulates the aggregate response intensity of each channel across the full time series. Subsequently, a dimensionality reduction linear layer W_*e*_, ReLU activation, and a restoration layer W_*r*_ jointly learn the weight distribution of each channel, which is then normalized via a sigmoid function to produce a gating signal s. Finally, this weight vector is broadcast-multiplied channel-wise with the original feature matrix H to generate the weighted feature representation H_*ca*_. This mechanism enables the model to autonomously prioritize frequency-domain features strongly associated with driving-induced fatigue, while attenuating noise and redundant channels, thereby enhancing both the discriminative power and physiological interpretability of the derived feature representations. The detailed computational procedures are presented in Equations 14–16.


$$z=\frac{1}{T}\,\overset{T}{\underset{t=1}{\sum }}h_{t}\in R^{2\,h}$$ (14)

$$s=\sigma \,\left(W_{r}\,\mathrm{ReLU}\,\left(W_{e}\,z\right)\right)\in R^{2\,h}\,\left(15\right)$$ (15)

$$H_{\mathrm{ca}}=H⊙s$$ (16)

Where: $W_{e}\in R^{\frac{2\,h}{r}\times 2\,h}$, $W_{r}\in R^{2\,h\times \frac{2\,h}{r}}$, compression ratio r = 16, ó(⋅) denotes the Sigmoid function, and⊙ represents broadcast multiplication along the channel dimension.

Temporal Attention(TA) focuses on capturing dynamic temporal structures within sequences by employing a compact neural network to generate learnable attention scores for each trial. These scores are normalized via the Softmax function and subsequently used to compute a weighted aggregation of the temporally calibrated feature representations. First, a two-layer perceptron applies nonlinear transformations to the channel-attention-calibrated feature $h_{t}^{c\,a}$ at each time step, producing raw attention scores *a_t_*. Subsequently, the Softmax function normalizes these scores into a probability distribution á_*t*_, ensuring that the sum of weights across all time steps equals 1. Finally, a global context-aware temporal aggregation feature vector *f*_*temp*_ is generated by performing a weighted sum of the hidden states. This mechanism enables the model to selectively attend to critical phases of fatigue accumulation, thereby enhancing its capability to discern non-stationary EEG patterns. The detailed computational procedures are presented in Equations 17–19.


$$a_{t}=w_{2}^{T}\,t\,a\,n\,h\,\left(W_{1}\,h_{t}^{\mathrm{ca}}+b_{1}\right)+b_{2}\in R$$ (17)

$$\alpha_{t}=\frac{\exp\,\left(a_{t}\right)}{\sum_{k=1}^{T}\exp\,\left(a_{k}\right)}$$ (18)

$$f_{\mathrm{temp}}=\overset{T}{\underset{t=1}{\sum }}\alpha_{t}\cdot h_{t}^{\mathrm{ca}}\in R^{2\,h}$$ (19)

Where: *W*_1_ ∈ *R*^32 × 2*h*^, *w*_2_ ∈ *R*^32^,α = [α_1_,…,α_*T*_] is the temporal attention weight.

## 4 Results and discussion

### 4.1 Evaluation metrics

To comprehensively evaluate the recognition performance and practical applicability of the model in the three-class fatigue-state classification task, this study adopted Accuracy, Balanced Accuracy, and Macro-F1 as evaluation metrics. Accuracy reflects the overall classification correctness, whereas Balanced Accuracy and Macro-F1 can alleviate the influence of class imbalance and are more suitable for assessing the model’s balanced recognition performance across the three states. The detailed computational procedures are presented in Equations 20–22.


$$\mathrm{Accuracy}=\frac{\sum_{c=1}^{N}{\mathrm{TP}}_{c}}{\sum_{c=1}^{N}\left({\mathrm{TP}}_{c}+{\mathrm{FN}}_{c}\right)}$$ (20)

$$B\,a\,l\,a\,n\,c\,e\,d\,A\,c\,c\,u\,r\,a\,c\,y=\frac{1}{N}\,\overset{N}{\underset{c=1}{\sum }}\frac{T\,P_{c}}{T\,P_{c}+F\,N_{c}}$$ (21)

$$M\,a\,c\,r\,o-F\,1=\frac{1}{N}\,\overset{N}{\underset{c=1}{\sum }}\frac{2\times T\,P_{c}}{2\times T\,P_{c}+F\,P_{c}+F\,N_{c}}$$ (22)

In addition, this study reports the number of parameters (M), model size (MB), and FLOPs(M) to evaluate the model’s parameter scale, storage overhead, and computational cost for single-sample inference.

### 4.2 Implementation details

All experiments were executed on a server operating under Ubuntu 22.04. The hardware configuration consisted of a high-performance computing node featuring a 16 vCPU Intel^®^ Xeon^®^ Gold 6430 processor, an NVIDIA RTX 4090 GPU, and 120 GB of system memory. The software environment was configured with Python 3.12 as the programming interpreter and PyTorch 2.3.0 as the deep learning framework, utilizing the CUDA 12.1 parallel computing architecture to accelerate model training. All experiments were conducted on this consistent computational platform to ensure the comparability and reproducibility of the results.

### 4.3 Behavioral and subjective validation of fatigue induction

To validate whether the task-stage-based labels reflected fatigue-related behavioral changes, we further analyzed subjective fatigue ratings and PVT behavioral performance before and after the 2-back fatigue induction task. The eight-level subjective fatigue scale was converted into numerical scores ranging from 1 to 8. As shown in Table 4, the subjective fatigue score significantly increased from 3.69 ± 1.89 before induction to 6.75 ± 0.86 after induction, indicating that participants experienced a higher level of perceived fatigue after the continuous 2-back task. Meanwhile, the total reaction time in PVT2 was significantly longer than that in PVT1, increasing from 7896.81 ± 2685.67 ms to 9339.56 ± 2738.01 ms, with an average increase of 1442.75 ± 765.93 ms. Moreover, all 16 participants showed longer total reaction times in PVT2 than in PVT1, indicating slower behavioral responses, reduced vigilance, and impaired sustained attention after fatigue induction. These behavioral and subjective results provide supporting evidence for defining PVT1, MID, and PVT2 as alert, intermediate, and fatigue states, respectively.

**TABLE 4:** Behavioral and subjective validation of fatigue induction.

Indicator	Before induction/PVT1	After induction/PVT2	Change	Statistical test	*p*-value
PVT total RT	7896.81 ± 2685.67 ms	9339.56 ± 2738.01 ms	+1442.75 ± 765.93 ms	Paired *t*-test	1.79 × 10^–6^
Subjective fatigue score	3.69 ± 1.89	6.75 ± 0.86	+3.06 ± 1.39	Wilcoxon signed-rank test	2.02 × 10^–4^

### 4.4 Experimental results of channel selection

Based on the nested LOSO feature selection procedure described above, the selection frequency of each feature was summarized across all 16 outer folds. As shown in Table 5, eight features were consistently selected in all LOSO folds, namely mean, rms, zero crossing rate, gamma power, beta power, std, var, and peak to elevance across different training-subject combinations. Among these features, mean, rms, and zero crossing rate showed extremely small median ANOVA *p*-values and high XGBoost inner-CV accuracies, suggesting strong statistical separability and classification capability. In addition, beta power and gamma power were also consistently retained across all folds, indicating that frequency-domain information contributed to fatigue-state discrimination under the cross-subject evaluation protocol. Overall, these results demonstrate that the selected feature subset was not derived from a single fixed data split, but showed stable relevance across the nested LOSO folds.

**TABLE 5:** Feature selection results.

Feature	Selection count	Selection frequency	Median ANOVA *p*-value	XGBoost inner-CV accuracy
Mean	16/16	1.000	< 1.0 × 10^–300^	0.9770 ± 0.0025
rms	16/16	1.000	< 1.0 × 10^–300^	0.9101 ± 0.0056
Zero crossing rate	16/16	1.000	< 1.0 × 10^–300^	0.8831 ± 0.0052
Gamma power	16/16	1.000	9.597 × 10^–234^	0.6650 ± 0.0078
Beta power	16/16	1.000	7.082 × 10^–139^	0.6580 ± 0.0062
std	16/16	1.000	< 1.0 × 10^–300^	0.6315 ± 0.0059
var	16/16	1.000	9.802 × 10^–107^	0.6312 ± 0.0059
Peak to peak	16/16	1.000	< 1.0 × 10^–300^	0.6251 ± 0.0060

**FIGURE 3:**
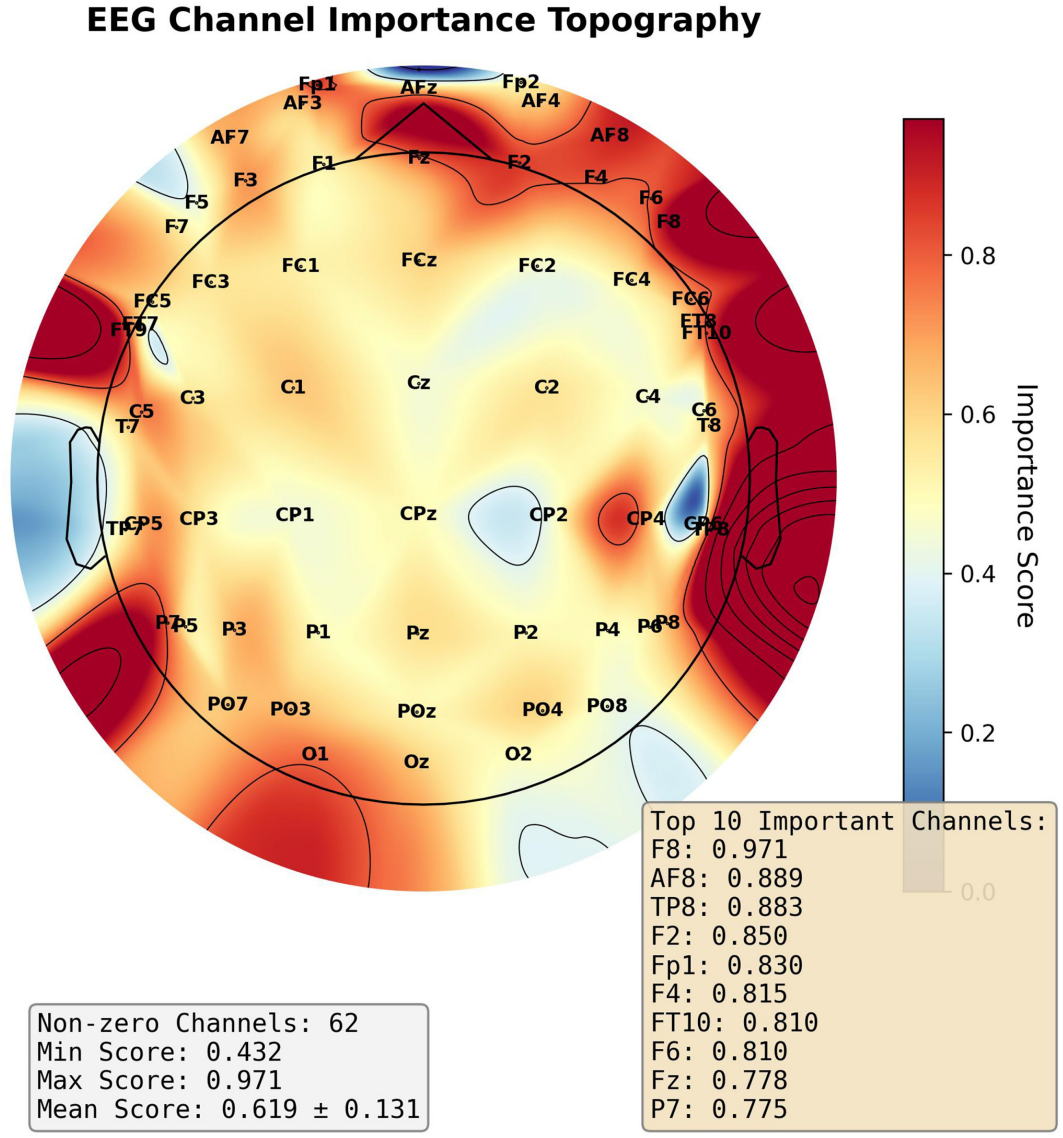
Channel importance topography.

To further analyze the spatial contribution of EEG channels to fatigue-state recognition, the composite importance score of each channel was calculated based on the sparse coefficients obtained from the L1-regularized logistic regression model within the nested LOSO framework. The score integrates the average absolute coefficient magnitude of features within each channel and the support ratio of non-zero coefficients, thereby jointly reflecting the contribution strength and selection stability of each EEG channel. After completing all 16 LOSO folds, the selection frequency and mean importance score of each channel were summarized to evaluate the stability of channel selection across different training-subject combinations. As shown in Figure 3, the spatial distribution of channel importance suggests that fatigue-related EEG information was mainly concentrated in the frontal, frontopolar, frontotemporal, parietal, and occipital regions. Table 6 further presents the stable EEG channels selected across the nested LOSO folds, together with their corresponding selection frequencies and mean importance scores. A total of 18 EEG channels were consistently selected in all 16 LOSO folds, including F8, AF8, TP8, F2, Fp1, F4, FT10, F6, Fz, P7, FT9, O1, C5, FT8, CP4, F3, FC6, and P3. Among these channels, F8 achieved the highest mean importance score, followed by AF8, TP8, F2, and Fp1, indicating that frontal and frontotemporal regions may play an important role in fatigue-state discrimination. In addition, the involvement of parietal and occipital channels suggests that fatigue recognition may also be associated with changes in sensorimotor integration and visual information processing. Overall, these results indicate that EEG-based fatigue detection relies on a distributed set of fatigue-related channels rather than a single localized brain region.

**TABLE 6:** Channel selection results under the nested LOSO protocol.

Channel	Selection count	Selection frequency	Mean L1-based importance score
F8	16/16	1.000	0.9711
AF8	16/16	1.000	0.8892
TP8	16/16	1.000	0.8826
F2	16/16	1.000	0.8503
Fp1	16/16	1.000	0.8296
F4	16/16	1.000	0.8154
FT10	16/16	1.000	0.8099
F6	16/16	1.000	0.8096
Fz	16/16	1.000	0.7783
P7	16/16	1.000	0.7746
FT9	16/16	1.000	0.7730
O1	16/16	1.000	0.7708
C5	16/16	1.000	0.7469
FT8	16/16	1.000	0.7218
CP4	16/16	1.000	0.7195
F3	16/16	1.000	0.7101
FC6	16/16	1.000	0.7088
P3	16/16	1.000	0.6617

### 4.5 Experimental comparison results of EfficientLSTM

In the model performance comparison experiments, the LOSO-CV strategy was adopted to evaluate EfficientLSTM and all baseline models. In each fold, one participant was used as the independent test subject, while the remaining 15 participants were used for training. Feature selection, channel selection, and model training were performed only within the training subjects of each fold. All models were evaluated using the same LOSO partitioning strategy and the same feature matrix settings. The final results are reported as the mean ± standard deviation across the 16 LOSO folds. In this study, a total of 19 time-frequency, spectral, and nonlinear features were extracted from the data and organized into a feature matrix A with dimensions 19 × 62 × 6,872. To enhance the feature selection process, dual screening was performed using ANOVA and XGBoost, leading to the selection of 8 features, which were subsequently arranged into a feature matrix B with dimensions 8 × 62 × 6,872. Finally, L1-regularized logistic-regression-based sparse channel selection was applied to further refine the channel set, resulting in the selection of 18 EEG channels. This produced a final feature matrix C with dimensions 8 × 18 × 6,872.

To ensure a fair and clear comparison between temporal and non-temporal models, the input format for each model category was explicitly defined. For temporal deep learning models, including GRU, LSTM, BiLSTM, TF_Transformer, 1D-CNN, and EfficientLSTM, consecutive EEG segments were organized into temporal sequences with a sequence length of 10. Each EEG segment was represented as a D-dimensional feature vector, where D was 19 × 62, 8 × 62, and 8 × 18 for feature matrices A, B, and C, respectively. Therefore, the temporal models received three-dimensional inputs with the shape N × 10 × D, enabling them to explicitly model sequence-level temporal dependencies across adjacent EEG segments. In particular, 1D-CNN preserved the sequence structure and applied temporal convolution along the time dimension, while TF_Transformer used positional encoding and self-attention to model temporal relationships. For non-temporal models, including XGBoost, KNN, SVM, LR, and MLP, the same temporal windows with a sequence length of 10 were first constructed to ensure that these models had access to the same temporal-window information. However, because these models do not explicitly process sequential inputs in the same manner as recurrent, convolutional, or Transformer-based temporal models, each temporal window was flattened into a two-dimensional feature vector before classification. For example, for feature matrix C, each EEG segment had D = 8 × 18 = 144 feature values, and each temporal-window sample was flattened from 10 × 144 into a 1,440-dimensional vector. Thus, the non-temporal models used the same temporal-window information as the deep sequential models, but they did not explicitly model temporal order or sequence-level dependencies. The performance of EfficientLSTM was compared with widely used classifiers, including GRU, XGBoost, TF_Transformer, 1D-CNN, KNN, SVM, LSTM, LR, MLP, and BiLSTM. Classification experiments were conducted on feature matrices A, B, and C, and the results are summarized in Tables 7–9.

**TABLE 7:** Classification performance of each model on feature set A.

Model	Accuracy	Balanced_ Accuracy	Macro_F1	Params(M)	Model Size (MB)	FLOPs (M)
GRU (Cho et al., 2014)	0.6008 ± 0.2210	0.6061 ± 0.2252	0.5721 ± 0.2423	0.2716	1.0360	5.3455
XGBOOST (Chen and Guestrin, 2016)	0.6579 ± 0.2611	0.6727 ± 0.2552	0.6214 ± 0.2675	0.00439	0.3599	0.00357
TF_Transformer (Lim et al., 2021)	0.6576 ± 0.1810	0.6614 ± 0.1843	0.6083 ± 0.2048	0.1789	0.6828	3.5824
1D-CNN (Malik et al., 2021)	0.6699 ± 0.1656	0.6739 ± 0.1693	0.6234 ± 0.2113	0.3127	1.1955	6.1014
KNN (Yigit, 2015)	0.5095 ± 0.1653	0.5196 ± 0.1651	0.4787 ± 0.1812	72.3992	276.2280	217.1976
SVM (Cortes and Vapnik, 1995)	0.6299 ± 0.1975	0.6289 ± 0.1968	0.5779 ± 0.2395	48.6292	371.1529	145.9284
LSTM (Graves, 2012)	0.6331 ± 0.2005	0.6361 ± 0.2040	0.5756 ± 0.2142	0.3607	1.3758	7.1245
LR (Zabor et al., 2022)	0.6572 ± 0.1813	0.6610 ± 0.1800	0.6251 ± 0.2139	0.0359	0.2746	0.0718
MLP (Rumelhart et al., 1985)	0.6462 ± 0.1951	0.6512 ± 0.1988	0.6110 ± 0.2272	3.1037	11.8424	6.2053
BiLSTM (Duyar et al., 2024)	0.6416 ± 0.1832	0.6439 ± 0.1855	0.6032 ± 0.2106	0.7538	2.8756	14.9039
EfficientLSTM	0.6713 ± 0.1791	0.6752 ± 0.1810	0.6316 ± 0.2086	0.9341	3.5635	18.4660

**TABLE 8:** Classification performance of each model on feature set B.

Model	Accuracy	Balanced_ Accuracy	Macro_F1	Params (M)	Model size (MB)	FLOPs (M)
GRU	0.6136 ± 0.2516	0.6199 ± 0.2522	0.5684 ± 0.2577	0.1387	0.5289	2.6870
XGBOOST	0.6745 ± 0.2594	0.6883 ± 0.2539	0.6380 ± 0.2663	0.00438	0.3582	0.00357
TF_Transformer	0.6509 ± 0.2135	0.6561 ± 0.2090	0.6168 ± 0.2183	0.1346	0.5138	2.6963
1D-CNN	0.6426 ± 0.2028	0.6441 ± 0.2043	0.5945 ± 0.2392	0.1797	0.6884	3.4429
KNN	0.5862 ± 0.2283	0.5895 ± 0.2323	0.5485 ± 0.2356	30.4839	116.3337	91.4516
SVM	0.6590 ± 0.2155	0.6581 ± 0.2146	0.6199 ± 0.2339	13.7658	105.1203	41.3248
LSTM	0.6684 ± 0.2282	0.6704 ± 0.2274	0.6499 ± 0.2332	0.1834	0.6997	3.5798
LR	0.6467 ± 0.2179	0.6505 ± 0.2156	0.5956 ± 0.2344	0.0151	0.1161	0.0302
MLP	0.6675 ± 0.2333	0.6672 ± 0.2335	0.6202 ± 0.2654	1.3313	5.0816	2.6607
BiLSTM	0.6457 ± 0.1894	0.6455 ± 0.1889	0.6044 ± 0.2082	0.3913	1.4927	7.7985
EfficientLSTM	0.6884 ± 0.2335	0.6916 ± 0.2310	0.6578 ± 0.2439	0.3239	1.2360	6.2666

**TABLE 9:** Classification performance of each model on feature set C.

Model	Accuracy	Balanced_ Accuracy	Macro_F1	Params (M)	Model Size (MB)	FLOPs (M)
GRU	0.7089 ± 0.2190	0.7090 ± 0.2196	0.6722 ± 0.2375	0.0655	0.2498	1.2983
XGBOOST	0.6968 ± 0.2533	0.7015 ± 0.2552	0.6447 ± 0.2617	0.0044	0.3608	0.0036
TF_Transformer	0.7356 ± 0.2072	0.7359 ± 0.2071	0.6964 ± 0.2273	0.1116	0.4260	2.2361
1D-CNN	0.7025 ± 0.2272	0.7005 ± 0.2260	0.6636 ± 0.2573	0.1107	0.4251	2.0624
KNN	0.5741 ± 0.2182	0.5775 ± 0.2224	0.5282 ± 0.2323	8.7183	33.3046	26.1549
SVM	0.7099 ± 0.1790	0.7088 ± 0.1782	0.6621 ± 0.2003	3.6885	28.2304	11.0910
LSTM	0.7311 ± 0.2241	0.7291 ± 0.2230	0.6878 ± 0.2603	0.0872	0.3328	1.7309
LR	0.7069 ± 0.1691	0.7111 ± 0.1689	0.6610 ± 0.1982	0.0043	0.0338	0.0087
MLP	0.7062 ± 0.2015	0.7073 ± 0.2026	0.6623 ± 0.2246	0.1933	0.7388	0.3856
BiLSTM	0.7383 ± 0.1728	0.7410 ± 0.1743	0.6985 ± 0.1982	0.2072	0.7905	4.1173
EfficientLSTM	0.7454 ± 0.2147	0.7436 ± 0.2139	0.7143 ± 0.2452	0.1959	0.7474	3.7069

Tables 7–9 report the classification performance of all compared models on feature matrices A, B, and C under the nested LOSO-CV protocol. Overall, the results indicate that the optimized feature-channel representation improved the cross-subject fatigue recognition performance of most models. On feature matrix A, which used the original 19 features from all 62 channels, the overall performance of the compared models was relatively limited, suggesting that the original high-dimensional representation may contain redundant or less discriminative information. After ANOVA-XGBoost feature screening and further L1-based channel selection, matrix C provided a more compact representation with dimensions of 8 × 18 and achieved the best overall results among the three feature matrices. It should be noted that several traditional machine learning models, such as XGBoost and LR, required fewer parameters and lower computational cost than EfficientLSTM, indicating their advantages in extremely lightweight deployment scenarios. However, their recognition performance, especially Macro-F1, was lower than that of EfficientLSTM under the LOSO protocol. In contrast, EfficientLSTM achieved the highest average Accuracy and Macro-F1 on the optimized feature matrix C, suggesting that the proposed model can better exploit temporal EEG patterns and selected fatigue-related feature-channel information. Therefore, compared with extremely lightweight traditional models, EfficientLSTM provides a more favorable balance between cross-subject recognition performance, temporal modeling capability, and model complexity.

Table 10 summarizes the performance of EfficientLSTM under the three feature-matrix settings using the nested LOSO-CV protocol. On the original feature matrix A, EfficientLSTM achieved an Accuracy of 0.6713 ± 0.1791, a Balanced Accuracy of 0.6752 ± 0.1810, and a Macro-F1 of 0.6316 ± 0.2086. After feature refinement using ANOVA and XGBoost, feature matrix B improved the performance to 0.6884 ± 0.2335 in Accuracy, 0.6916 ± 0.2310 in Balanced Accuracy, and 0.6578 ± 0.2439 in Macro-F1. Further channel optimization using L1-based channel selection produced the best overall performance on feature matrix C, with an Accuracy of 0.7454 ± 0.2147, a Balanced Accuracy of 0.7436 ± 0.2139, and a Macro-F1 of 0.7143 ± 0.2452. Compared with matrix A, matrix C improved Accuracy, Balanced Accuracy, and Macro-F1 by 7.41, 6.84, and 8.27 percentage points, respectively. Compared with matrix B, matrix C further improved these three metrics by 5.70, 5.20, and 5.65 percentage points, respectively. Meanwhile, the computational complexity of EfficientLSTM was substantially reduced as the feature-channel representation became more compact. From matrix A to matrix C, the number of parameters decreased from 0.9341 M to 0.1959 M, the model size decreased from 3.5635 MB to 0.7474 MB, and the FLOPs decreased from 18.4660 M to 3.7069 M. These results indicate that the progressive feature and channel selection strategy improves the cross-subject recognition performance of EfficientLSTM while simultaneously reducing model complexity. However, the relatively large standard deviations also suggest noticeable inter-subject variability, highlighting the challenge of EEG-based fatigue detection under strict LOSO validation.

**TABLE 10:** Classification performance of EfficientLSTM on feature sets A, B, C.

Feature matrix	Accuracy	Balanced_ Accuracy	Macro_F1	Params (M)	Model Size (MB)	FLOPs (M)
A	0.6713 ± 0.1791	0.6752 ± 0.1810	0.6316 ± 0.2086	0.9341	3.5635	18.4660
B	0.6884 ± 0.2335	0.6916 ± 0.2310	0.6578 ± 0.2439	0.3239	1.2360	6.2666
**C**	0.7454 ± 0.2147	0.7436 ± 0.2139	0.7143 ± 0.2452	0.1959	0.7474	3.7069

**FIGURE 4:**
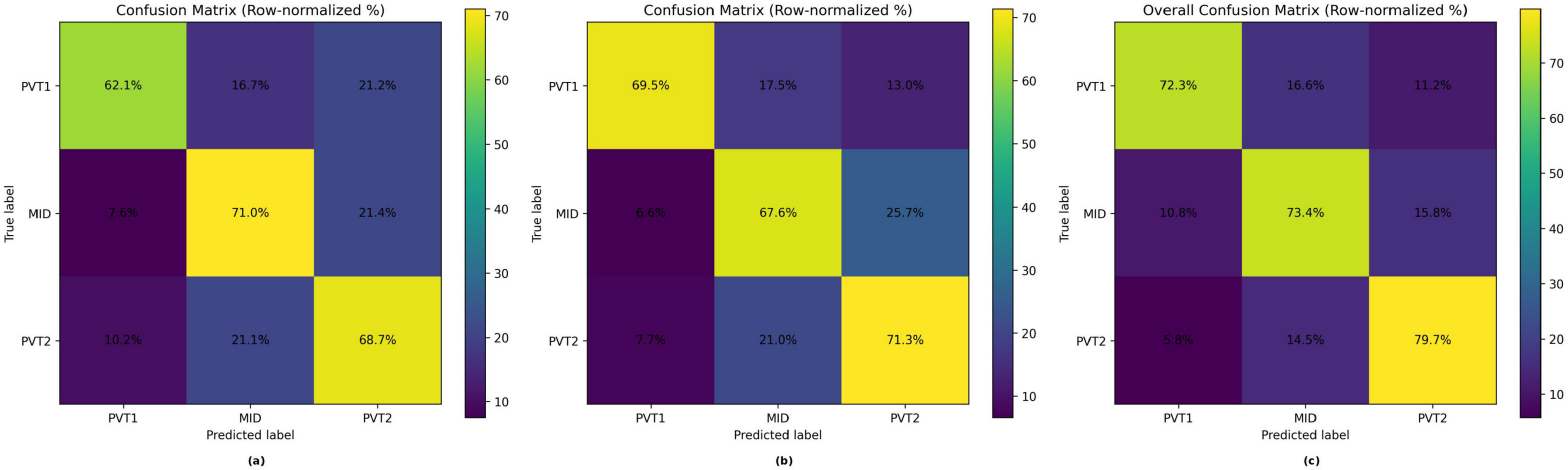
Confusion matrix of EfficientLSTM on the dataset. **(a)** Feature matrix A. **(b)** Feature matrix B. **(c)** Feature matrix C.

Figure 4 presents the row-normalized aggregated LOSO confusion matrices of EfficientLSTM on feature matrices A, B, and C. For matrix A, the recognition rates of PVT1, MID, and PVT2 were 62.1, 71.0, and 68.7%, respectively. After feature refinement, matrix B improved the recognition rates of PVT1 and PVT2 to 69.5 and 71.3%, while MID slightly decreased to 67.6%. With further channel optimization, matrix C achieved the best overall performance, with recognition rates of 72.3, 73.4, and 79.7% for PVT1, MID, and PVT2, respectively. These results indicate that the optimized feature-channel subset improved class separability and enhanced the recognition of fatigue states under the LOSO setting.

### 4.6 Pairwise statistical significance analysis under the LOSO protocol

To further evaluate whether the performance differences between EfficientLSTM and the baseline models were statistically significant, pairwise Wilcoxon signed-rank tests were conducted based on the per-subject LOSO Macro-F1 scores. The Holm–Bonferroni correction was applied to adjust the *p*-values for multiple comparisons. As shown in Table 11, EfficientLSTM achieved positive mean differences in Macro-F1 compared with all baseline models, indicating that it obtained the highest average Macro-F1 under the LOSO protocol. In particular, EfficientLSTM outperformed KNN in all 16 LOSO folds, with a mean Macro-F1 improvement of 0.1861, and this difference remained statistically significant after correction *p*_*adj*_=3.05×10^−4^. However, after Holm–Bonferroni correction, the differences between EfficientLSTM and several competitive baseline models did not reach statistical significance. This result may be attributed to the substantial inter-subject variability under the LOSO protocol and the relatively close performance among top-performing temporal models. Nevertheless, EfficientLSTM achieved the highest average Macro-F1 among all compared models and showed positive mean differences against all baselines, suggesting that it provides a competitive and robust cross-subject fatigue recognition performance.

**TABLE 11:** Pairwise Wilcoxon signed-rank test results between EfficientLSTM and baseline models under the LOSO protocol.

Comparison	Mean difference	Win/Tie/Loss	*p*-value	Adjusted *p*-value	Significance
EfficientLSTM vs. KNN	0.1861	16/0/0	3.05 × 10^–5^	3.05 × 10^–4^	***
EfficientLSTM vs. GRU	0.0422	12/0/4	0.0335	0.3018	ns
EfficientLSTM vs. 1D-CNN	0.0507	11/0/5	0.0507	0.4053	ns
EfficientLSTM vs. LR	0.0534	11/0/5	0.1297	0.6541	ns
EfficientLSTM vs. MLP	0.0520	11/0/5	0.0934	0.6541	ns
EfficientLSTM vs. LSTM	0.0265	10/1/5	0.0995	0.6541	ns
EfficientLSTM vs. SVM	0.0522	11/0/5	0.1439	0.6541	ns
EfficientLSTM vs. BiLSTM	0.0159	10/0/6	0.5282	1.0000	ns
EfficientLSTM vs. TF_Transformer	0.0179	10/0/6	0.4332	1.0000	ns
EfficientLSTM vs. XGBoost	0.0696	8/0/8	0.3755	1.0000	ns

Pairwise comparisons were performed using the Wilcoxon signed-rank test based on per-subject LOSO Macro-F1 scores. Adjusted *p*-values were obtained using the Holm–Bonferroni correction. Win/Tie/Loss indicates the number of LOSO folds in which EfficientLSTM achieved higher, equal, or lower Macro-F1 than the corresponding baseline model.

*** indicates adjusted *p* < 0.001, and ns indicates no statistical significance.

### 4.7 Computational efficiency-performance trade-off and sensitivity analysis

To further evaluate the trade-off between recognition performance and computational efficiency, a computational efficiency–performance analysis was conducted under the LOSO protocol. Since XGBoost achieved the lowest FLOPs among all compared models, it was used as the computational baseline. For each model, the relative FLOPs were calculated as the ratio between its FLOPs and the FLOPs of XGBoost. In addition, the performance gains were quantified by calculating the differences in Accuracy and Macro-F1 relative to XGBoost. As shown in Table 12, XGBoost required only 0.0036M FLOPs, indicating its clear advantage in computational efficiency. However, under the stricter LOSO evaluation protocol, EfficientLSTM achieved higher recognition performance than XGBoost. Specifically, EfficientLSTM improved Accuracy from 0.6968 to 0.7454 and Macro-F1 from 0.6447 to 0.7143, corresponding to gains of 0.0486 and 0.0696, respectively. These results indicate that EfficientLSTM provides better cross-subject fatigue recognition performance, although it requires a higher computational cost.

**TABLE 12:** Computational efficiency-performance trade-off analysis relative to XGBoost under the LOSO protocol.

Model	Accuracy	Macro-F1	FLOPs (M)	Relative FLOPs vs. XGBoost	ΔAccuracy vs. XGBoost	ΔMacro-F1 vs. XGBoost
GRU	0.7089	0.6722	1.2983	360.64 ×	0.0121	0.0275
XGBoost	0.6968	0.6447	0.0036	1.00 ×	0.0000	0.0000
TF_Transformer	0.7356	0.6964	2.2361	621.14 ×	0.0388	0.0517
1D-CNN	0.7025	0.6636	2.0624	572.89 ×	0.0057	0.0189
KNN	0.5741	0.5282	26.1549	7265.25 ×	−0.1227	−0.1165
SVM	0.7099	0.6621	11.0910	3080.83 ×	0.0131	0.0174
LSTM	0.7311	0.6878	1.7309	480.81 ×	0.0343	0.0431
LR	0.7069	0.6610	0.0087	2.42 ×	0.0101	0.0163
MLP	0.7062	0.6623	0.3856	107.11 ×	0.0094	0.0176
BiLSTM	0.7383	0.6985	4.1173	1143.69 ×	0.0415	0.0538
EfficientLSTM	0.7454	0.7143	3.7069	1029.69 ×	0.0486	0.0696

XGBoost was used as the computational baseline because it achieved the lowest FLOPs among all compared models. Relative FLOPs were calculated as the ratio between each model’s FLOPs and XGBoost FLOPs. ΔAccuracy and ΔMacro-F1 denote the performance differences relative to XGBoost.

Among the temporal deep learning models, EfficientLSTM achieved the highest Macro-F1 while using fewer parameters, a smaller model size, and lower FLOPs than BiLSTM. This suggests that EfficientLSTM does not simply rely on increased model complexity to improve performance, but achieves a favorable balance among cross-subject recognition performance, temporal modeling capability, and computational cost. Although lightweight models such as XGBoost and LR have clear advantages in extremely low-computation scenarios, EfficientLSTM is more suitable for fatigue detection applications that require a balance between recognition performance, model lightweight design, and sequence-level EEG temporal feature modeling.

### 4.8 Ablation study results of EfficientLSTM

To validate the sensitivity of EfficientLSTM to key hyperparameters, this study investigated the effects of the number of hidden units, dropout rate, learning rate, and feature compression ratio on model performance. The experimental results are shown in Table 13. An appropriate increase in the number of hidden units can enhance the model’s capability to represent temporal features. However, when the number of hidden units is further increased to 96 or 128, the model performance decreases, indicating that an excessively large hidden dimension may introduce redundant parameters and increase the risk of overfitting. Meanwhile, variations in dropout rate, learning rate, and compression ratio also exert a noticeable influence on model performance. A relatively low dropout rate may be insufficient to effectively mitigate overfitting, whereas an excessively small learning rate may hinder model convergence. In addition, an overly high compression ratio may lead to the loss of discriminative information.

**TABLE 13:** Hyperparameter sensitivity analysis.

Hyperparameter					Evaluation metrics		
Set	Hidden units	Dropout	Learning rate	Compression ratio	Accuracy	Balanced_ Accuracy	Macro_F1
1	32	0.3	0.001	2	0.7445 ± 0.2159	0.7429 ± 0.2155	0.6920 ± 0.2432
2	**64**	**0.3**	**0.001**	**2**	**0.7454 ± 0.2147**	**0.7436 ± 0.2139**	**0.7143 ± 0.2452**
3	96	0.3	0.001	2	0.7148 ± 0.2563	0.7172 ± 0.2574	0.6869 ± 0.2778
4	128	0.3	0.001	2	0.7313 ± 0.1860	0.7333 ± 0.1870	0.7002 ± 0.2066
5	64	0.2	0.001	2	0.7428 ± 0.1891	0.7456 ± 0.1905	0.7086 ± 0.2215
6	64	0.1	0.001	2	0.7132 ± 0.2415	0.7151 ± 0.2428	0.6834 ± 0.2544
7	64	0.3	0.0001	2	0.7274 ± 0.2179	0.7263 ± 0.2173	0.6866 ± 0.2526
8	64	0.3	0.001	4	0.7272 ± 0.2325	0.7245 ± 0.2309	0.7028 ± 0.2419

Based on the overall experimental results, the hyperparameter combination of 64 hidden units, a dropout rate of 0.3, a learning rate of 0.001, and a compression ratio of 2 achieved the best performance, with Accuracy, Balanced_Accuracy and Macro_F1 reaching 0.7454 ± 0.2147, 0.7436 ± 0.2139 and 0.7143 ± 0.2452, respectively. Therefore, this hyperparameter setting was adopted as the optimal configuration for EfficientLSTM in this study. These results indicate that a moderate model capacity, appropriate regularization strength, suitable learning rate, and moderate feature compression can achieve a favorable balance among feature representation, information preservation, and generalization capability.

### 4.9 Ablation study results of EfficientLSTM

To assess the effectiveness of the EfficientLSTM model, ablation experiments were conducted by sequentially incorporating the model compression module (FCM), BiLSTM, channel attention (CA), and temporal attention (TA). These experiments were performed on the selected feature matrix C with dimensions 8 × 18 × 6,872, and the results are summarized in Table 14.

**TABLE 14:** Component ablation study of EfficientLSTM.

Model	Accuracy	Balanced_ Accuracy	Macro_F1	Params (M)	Model Size (MB)	FLOPs (M)
LSTM	0.7311 ± 0.2241	0.7291 ± 0.2230	0.6878 ± 0.2603	0.0872	0.3328	1.7309
FCM+LSTM	0.7306 ± 0.2290	0.7298 ± 0.2286	0.6913 ± 0.2684	0.0840	0.3209	1.6262
FCM+LSTM+CA	0.6770 ± 0.2800	0.6755 ± 0.2790	0.6354 ± 0.2920	0.0846	0.3231	1.6285
FCM+LSTM+TA	0.7264 ± 0.1977	0.7266 ± 0.1987	0.6874 ± 0.2270	0.0871	0.3327	1.6525
FCM+LSTM+CA+TA	0.6862 ± 0.2833	0.6867 ± 0.2834	0.6531 ± 0.3005	0.0877	0.3349	1.6548
LSTM+CA	0.6755 ± 0.2374	0.6767 ± 0.2378	0.6242 ± 0.2564	0.0899	0.3431	1.7372
LSTM+TA	0.6564 ± 0.2471	0.6606 ± 0.2483	0.6175 ± 0.2564	0.0924	0.3527	1.7612
LSTM+CA+TA	0.6760 ± 0.2315	0.6778 ± 0.2311	0.6320 ± 0.2511	0.0930	0.3549	1.7635
BiLSTM	0.7383 ± 0.1728	0.7410 ± 0.1743	0.6985 ± 0.1982	0.2072	0.7905	4.1173
FCM+BiLSTM	0.6837 ± 0.2446	0.6817 ± 0.2434	0.6440 ± 0.2556	0.1854	0.7076	3.6066
FCM+BiLSTM+CA	0.7321 ± 0.2397	0.7300 ± 0.2388	0.7104 ± 0.2494	0.1876	0.7159	3.6121
FCM+BiLSTM+TA	0.7337 ± 0.2270	0.7311 ± 0.2256	0.6961 ± 0.2605	0.1937	0.7391	3.7002
BiLSTM+CA	0.6926 ± 0.1724	0.6943 ± 0.1708	0.7007 ± 0.2030	0.2133	0.8140	4.1316
BiLSTM+TA	0.7061 ± 0.2512	0.7096 ± 0.2514	0.6706 ± 0.2673	0.2194	0.8372	4.2186
BiLSTM+CA+TA	0.7022 ± 0.2043	0.7041 ± 0.2065	0.6694 ± 0.2107	0.2216	0.8455	4.2252
FCM+BiLSTM+CA+TA (EfficientLSTM)	0.7454 ± 0.2147	0.7436 ± 0.2139	0.7143 ± 0.2452	0.1959	0.7474	3.7069

To evaluate the contribution of each component in EfficientLSTM, ablation experiments were conducted on the refined feature matrix C with dimensions of (8 × 18 × 6,872). As shown in Table 8, the complete EfficientLSTM achieved the best overall performance among all variants, with an Accuracy of 0.7454 ± 0.2147, a Balanced Accuracy of 0.7436 ± 0.2139, and a Macro-F1 of 0.7143 ± 0.2452. Compared with the baseline LSTM, EfficientLSTM improved Accuracy, Balanced Accuracy, and Macro-F1 from 0.7311 ± 0.2241, 0.7291 ± 0.2230, and 0.6878 ± 0.2603 to 0.7454 ± 0.2147, 0.7436 ± 0.2139, and 0.7143 ± 0.2452, respectively, indicating improved cross-subject fatigue recognition performance. The results further show that the contribution of each module depends on the model backbone. FCM reduced model complexity in both unidirectional and bidirectional settings, but FCM alone did not always improve recognition performance, suggesting that excessive feature compression may discard useful discriminative information. Similarly, directly adding CA or TA to LSTM did not consistently improve performance, whereas combining attention modules with FCM and BiLSTM produced more favorable results. For example, FCM+BiLSTM+CA achieved a Macro-F1 of 0.7104 ± 0.2494, higher than FCM+BiLSTM. Compared with BiLSTM, EfficientLSTM achieved higher Accuracy and Macro-F1 while reducing parameters from 0.2072 M to 0.1959 M, model size from 0.7905 MB to 0.7474 MB, and FLOPs from 4.1173 M to 3.7069 M. These results demonstrate that EfficientLSTM improves recognition performance not by simply stacking modules, but by jointly integrating feature compression, bidirectional temporal modeling, and attention-based recalibration to achieve a better balance between accuracy and computational efficiency.

### 4.10 SHAP-based interpretability analysis

**FIGURE 5:**
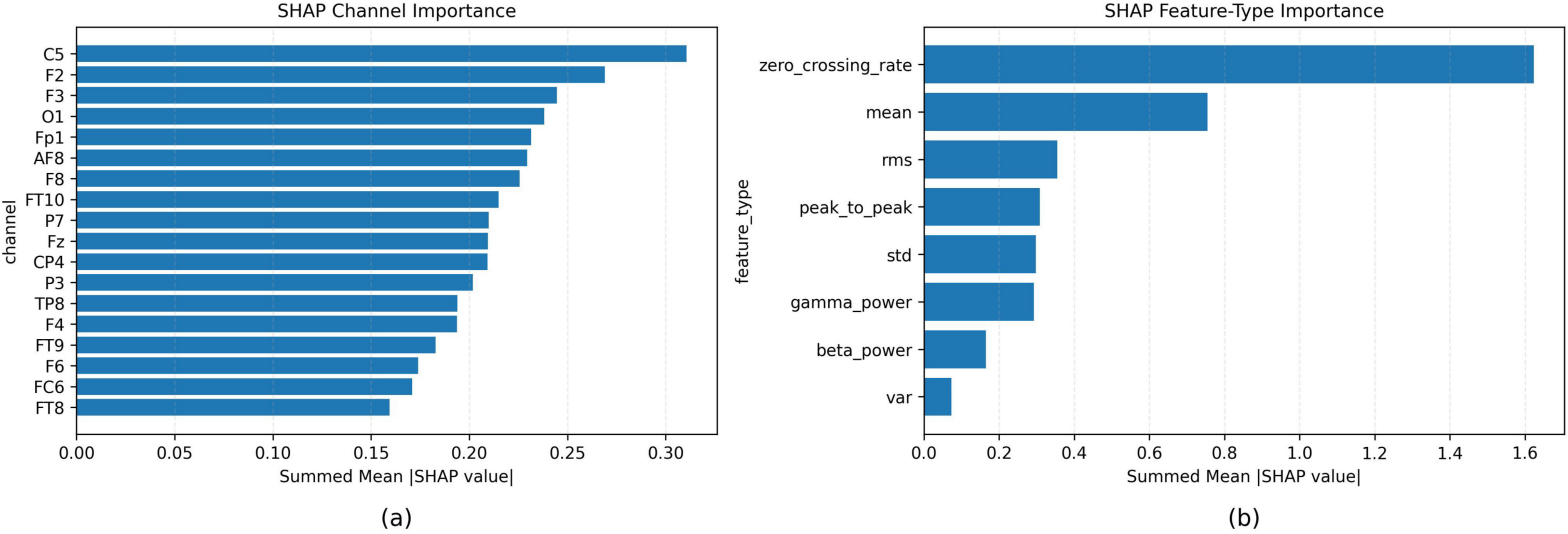
SHAP-based channel and feature-type importance analysis. **(a)** Channel-level importance ranking based on the summed mean absolute SHAP values. **(b)** Feature-type importance ranking based on the summed mean absolute SHAP values.

To further investigate the decision-making mechanism of EfficientLSTM under the LOSO protocol, SHAP analysis was performed on the selected feature-channel subset (Lundberg and Lee, 2017). As shown in Figure 5a, the channel-level SHAP results indicate that different EEG channels contributed unequally to the model predictions. Among the selected channels, C5, F2, F3, O1, Fp1, AF8, F8, FT10, P7, Fz, CP4, P3, and TP8 showed relatively high summed mean absolute SHAP values. These channels are distributed across frontal, frontopolar, frontotemporal, central, parietal, and occipital regions, suggesting that fatigue-related EEG information is reflected in distributed cortical activity rather than a single localized area. The relatively high contribution of C5 may be associated with central cortical activity related to response preparation and sensorimotor processing, which is relevant to the PVT and 2-back tasks requiring continuous attention and motor responses. Previous sustained-attention studies have reported that EEG features from left central and temporal regions can predict slower reaction times and vigilance variability, supporting the relevance of central-region EEG activity to fatigue-related performance decline (Torkamani-Azar et al., 2020). The importance of F2 and AF8 may reflect the involvement of frontal and frontopolar regions in executive control, attention regulation, and vigilance maintenance. This interpretation is consistent with previous studies showing that frontal or forehead EEG channels can provide useful information for detecting driver fatigue and vigilance decline (Yang et al., 2025). In addition, the contribution of TP8 may be related to temporoparietal activity associated with sustained attention, visuospatial processing, and fatigue-related cognitive state changes. Prior EEG fatigue studies have reported that temporal, parietal, temporoparietal, and occipital channels, such as T8, P8, and O1, contain discriminative information for fatigue recognition, which is broadly consistent with the observed importance of TP8, P7, P3, and O1 in this study (Liu et al., 2020). Figure 5b shows the feature-type importance results. The zero-crossing rate was the most important feature, followed by mean, rms, peak-to-peak value, standard deviation, gamma power, beta power, and variance. This indicates that EfficientLSTM mainly relied on signal fluctuation characteristics, while also integrating amplitude-related statistical features and spectral information for fatigue recognition. It should be noted that SHAP values represent model-based feature contributions rather than direct causal neural mechanisms; therefore, the importance of these channels should be interpreted as predictive relevance rather than neurophysiological causality.

**FIGURE 6:**
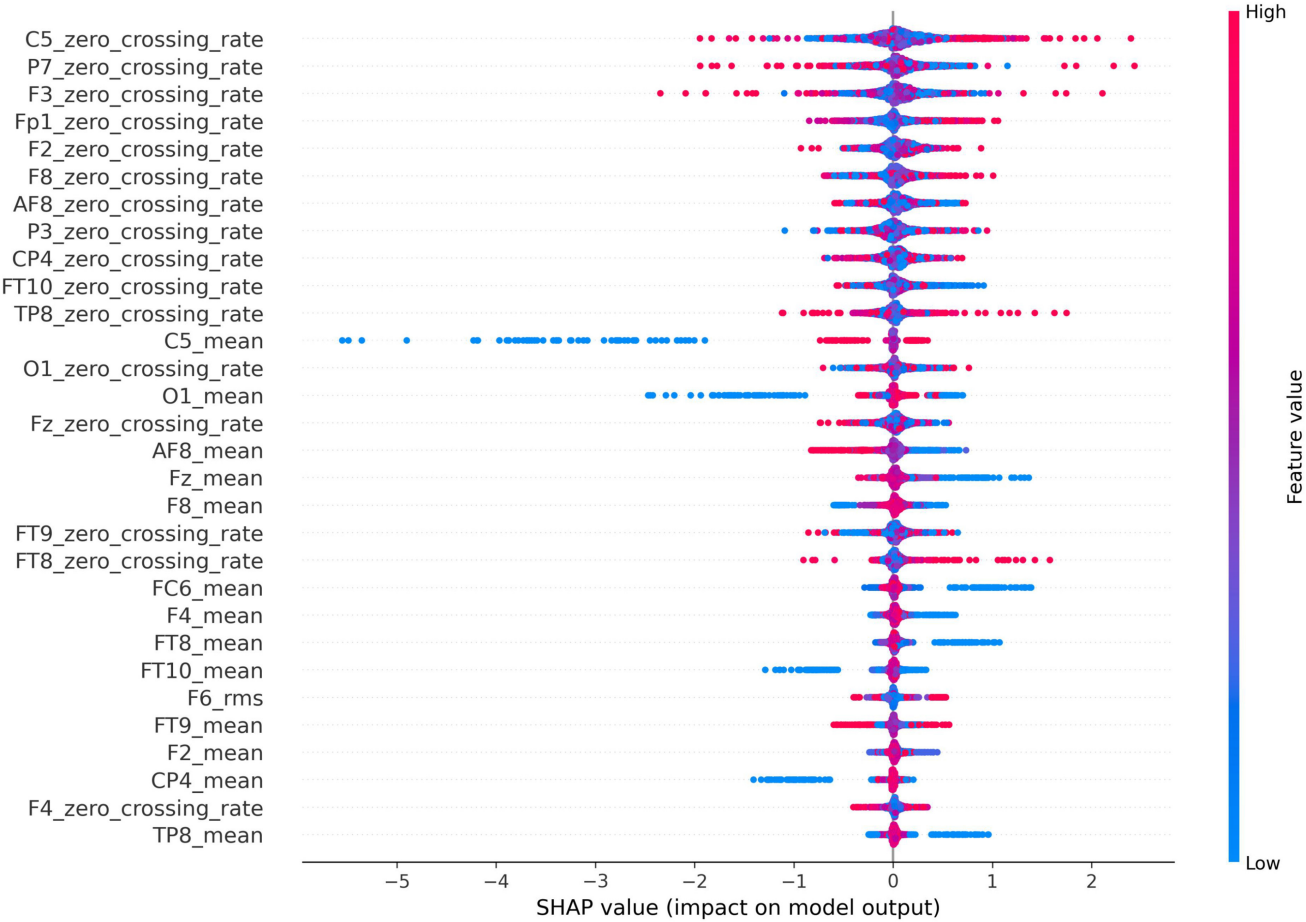
SHAP summary plot of top-ranked features.

Figure 6 further illustrates the effects of top-ranked feature-channel combinations on model outputs. Zero-crossing-rate features from multiple channels dominated the important feature list, confirming the importance of dynamic EEG fluctuation patterns. Several mean-related features also contributed to the predictions, suggesting that amplitude-level information provided complementary evidence. Overall, the LOSO-based SHAP results indicate that EfficientLSTM integrates information from multiple EEG channels and heterogeneous feature types for cross-subject fatigue recognition. It should be noted that SHAP values represent model-based feature contributions rather than direct causal neural mechanisms.

## 5 Conclusion

This paper proposes AXELLSTM, an EEG-based fatigue recognition framework that integrates interpretable feature-channel selection with efficient temporal modeling. The framework leverages EEG signal features to identify fatigue states under a cross-subject evaluation setting. The acquired EEG data were first preprocessed in MATLAB, and 19 time-domain, frequency-domain, and nonlinear features were extracted. To avoid potential data leakage, all feature selection, channel selection, preprocessing parameter estimation, and model training procedures were embedded within a nested leave-one-subject-out cross-validation framework. In each LOSO fold, ANOVA and XGBoost-based feature evaluation were performed only on the training subjects, leading to the stable selection of eight fatigue-related features. These features were subsequently organized into channel-specific subsets, and an L1-regularized sparse selection model was applied to estimate channel importance, resulting in 18 EEG channels that were consistently retained across all LOSO folds. Subsequently, an efficient fatigue recognition model, termed EfficientLSTM, was designed by integrating feature compression, BiLSTM, and channel-temporal attention mechanisms. Classification experiments were performed using EfficientLSTM on three distinct feature matrices: the original (19 × 62 × 6,872)-dimensional matrix A, the feature-screened (8 × 62 × 6,872)-dimensional matrix B, and the further channel-optimized (8 × 18 × 6,872)-dimensional matrix C. Progressive refinement of features and channels led to consistent improvements in classification performance, with Macro-F1 increasing from 0.6316 ± 0.2086 on matrix A to 0.6578 ± 0.2439 on matrix B and further to 0.7143 ± 0.2452 on matrix C. These findings demonstrate that the proposed feature-channel screening strategy can preserve discriminative fatigue-related information while reducing redundant EEG representations and computational complexity. Furthermore, comparative experiments with representative traditional machine learning classifiers and deep sequential models showed that EfficientLSTM achieved the highest average Macro-F1 under the LOSO protocol, with an Accuracy of 0.7454 ± 0.2147, a Balanced Accuracy of 0.7436 ± 0.2139, and a Macro-F1 of 0.7143 ± 0.2452. In particular, compared with the standard BiLSTM baseline, EfficientLSTM achieved better average recognition performance while requiring fewer parameters, a smaller model size, and lower FLOPs. These results suggest that AXELLSTM provides a competitive and computationally efficient solution for cross-subject EEG-based fatigue recognition.

## Data Availability

The raw data supporting the conclusions of this article will be made available by the authors, without undue reservation.
